# Infants on the move: bibliometric analyses of observational vs. digital means of screening infant development

**DOI:** 10.3389/fnint.2023.1251252

**Published:** 2024-01-25

**Authors:** Hannah Varkey, Ha Phan, Phyllis Kittler, Anne Gordon, Elizabeth B. Torres

**Affiliations:** ^1^Psychology Department, Rutgers University, Piscataway, NJ, United States; ^2^New York State Institute for Basic Research in Developmental Disabilities/OPWDD, Institute for Basic Research in Developmental Disabilities (IBR), Staten Island, NY, United States; ^3^Rutgers University Center for Cognitive Science, Piscataway, NJ, United States; ^4^Rutgers Computational Biomedicine Imaging and Modeling, Piscataway, NJ, United States

**Keywords:** bibliometrics, infant development, general movements assessment, digital phenotyping, motor development

## Abstract

Neurodevelopmental disorders are on the rise, yet their average diagnosis is after 4.5 years old. This delay is partly due to reliance on social-communication criteria, which require longer maturation than scaffolding elements of neuromotor control. Much earlier criteria could include reflexes, monitoring of the quality of spontaneous movements from central pattern generators and maturation of intentional movements and their overall sensation. General Movement Assessment (GMA) studies these features using observational means, but the last two decades have seen a surge in digital tools that enable non-invasive, continuous tracking of infants’ spontaneous movements. Despite their importance, these tools are not yet broadly used. In this work, using CiteSpace, VOSViewer and SciMAT software, we investigate the evolution of the literature on GMA and the methods in use today, to estimate how digital techniques are being adopted. To that end, we created maps of key word co-occurrence networks, co-author networks, document co-citation analysis and strategic diagrams of 295 publications based on a search in the Web of Science, Dimensions and SCOPUS databases for: ‘general movement assessment’ OR ‘general movements assessment’. The nodes on the maps were categorized by size, cluster groups and year of publication. We found that the state-of-the-art methodology to diagnose neurodevelopmental disorders still relies heavily on observation. Several groups in classical GMA research have branched out to incorporate new techniques, but few groups have adopted digital means. We report on additional analyses of methods and biosensors usage and propose that combining traditional clinical observation criteria with digital means may allow earlier diagnoses and interventional therapies for infants.

## Introduction

As altricial mammals, humans require a long time to mature and reach social milestones (Garwicz et al., 2009). Yet, at birth, primitive structures of the human neonate’s brain, such as the brainstem, are mature enough to integrate disparate forms of sensory input, control central pattern generators, mediate reflexive behaviors and enable the neonate’s survival (Grillner and El Manira, 2020). Breathing, feeding, and excreting, along with crying, coughing, sneezing and other sensory-motor functions, offer a window into the intactness of the baby’s neurodevelopment far earlier than the social criteria currently in use (Torres et al., 2023). Indeed, observing and measuring biorhythmic activities in such early structures as infants explore the world around them can provide insight into the development of their nascent nervous systems.

**Figure 1:**
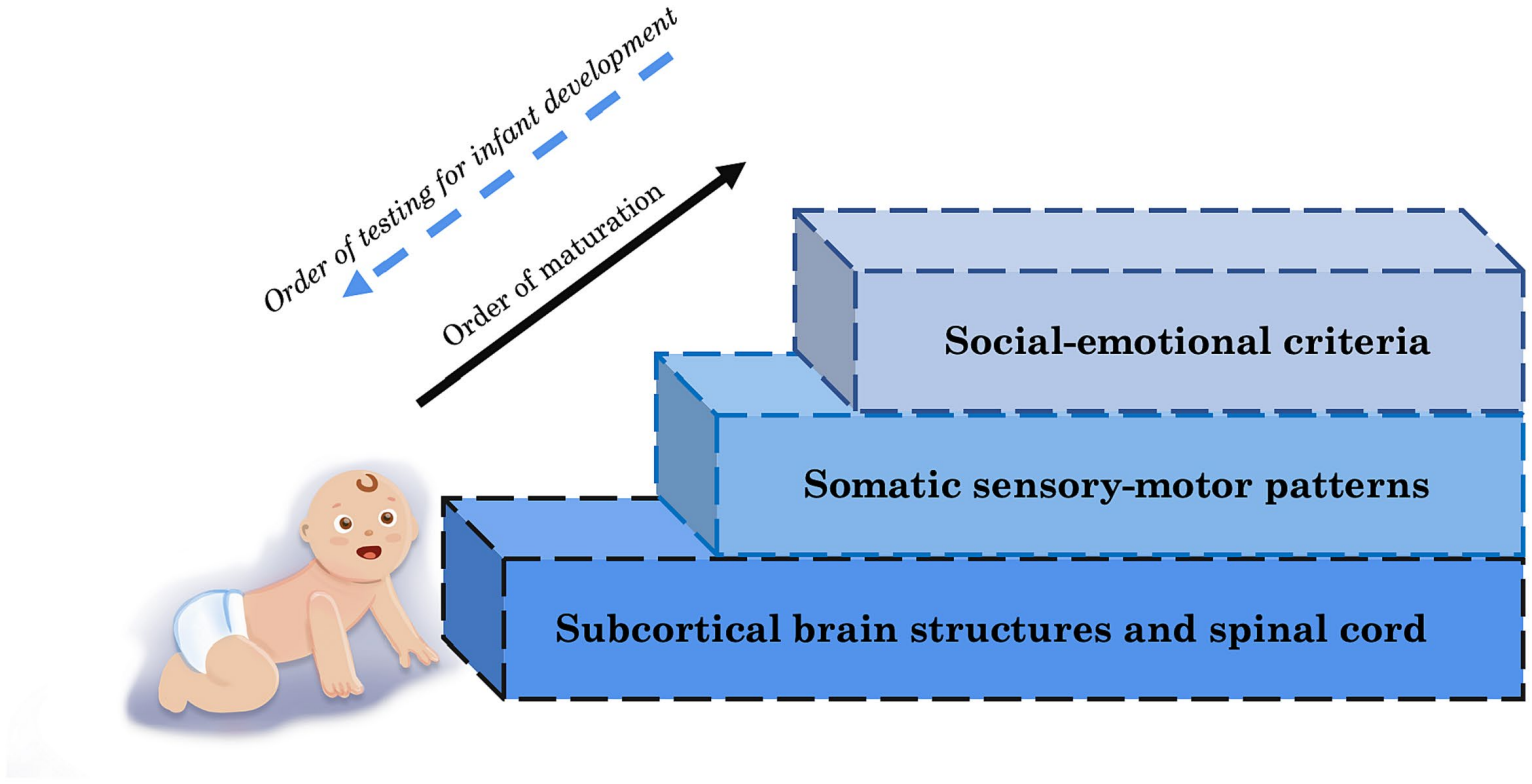
Levels of maturation for infant neurodevelopment whereby basic foundational building blocks of higher social levels necessarily mature first and are thus tractable and reliably detectable much earlier than current social–emotional-communicational criteria.

The means through which this insight is gained can vary—observational clinical tests based on social–emotional criteria, recording milestones of child development, and checking the presence of reflexes have been common measures of typical development, relying heavily on human observation of external behavior. Social communication and interaction of infants are aspects of development that form in the months after the scaffolding elements of neuromotor control mature. Digital technology like biosensors and digital biomarkers (Torres et al., 2016) can be used to investigate the ontogenetic progress of the nervous system such as auditory brainstem responses to assess brainstem function and the intactness of auditory neural pathways (Torres et al., 2023). Other methods include magnetic resonance imaging and ultrasound imaging (Gerall et al., 2023) as well as electroencephalography (Soul et al., 2023). Focusing on the foundation of nervous system development, the earliest layer of subcortical brain structures (shown in Figure 1) such as the cerebellum and basal ganglia, is instrumental in discovering neurodevelopmental derailment earlier to provide timely support or intervention (Torres et al., 2023).

As early as 8 to 10 weeks of gestation, a fetus possesses spontaneous, rhythmic, whole-body movements that are called ‘general movements’ (GMs; Prechtl, 1977; Prechtl, 1984). These movements may vary in amplitude, force, direction and speed, and their underlying neural circuitry involves central pattern generators located in the spinal cord and basal ganglia (Grillner and El Manira, 2020). The motor output can be seen without the presence of sensory input. The fetal movements may be simply observed through ultrasound recording, though this technique is not yet used to make conclusive findings in the clinical setting (Einspieler et al., 2012, 2021).

**Figure 2:**
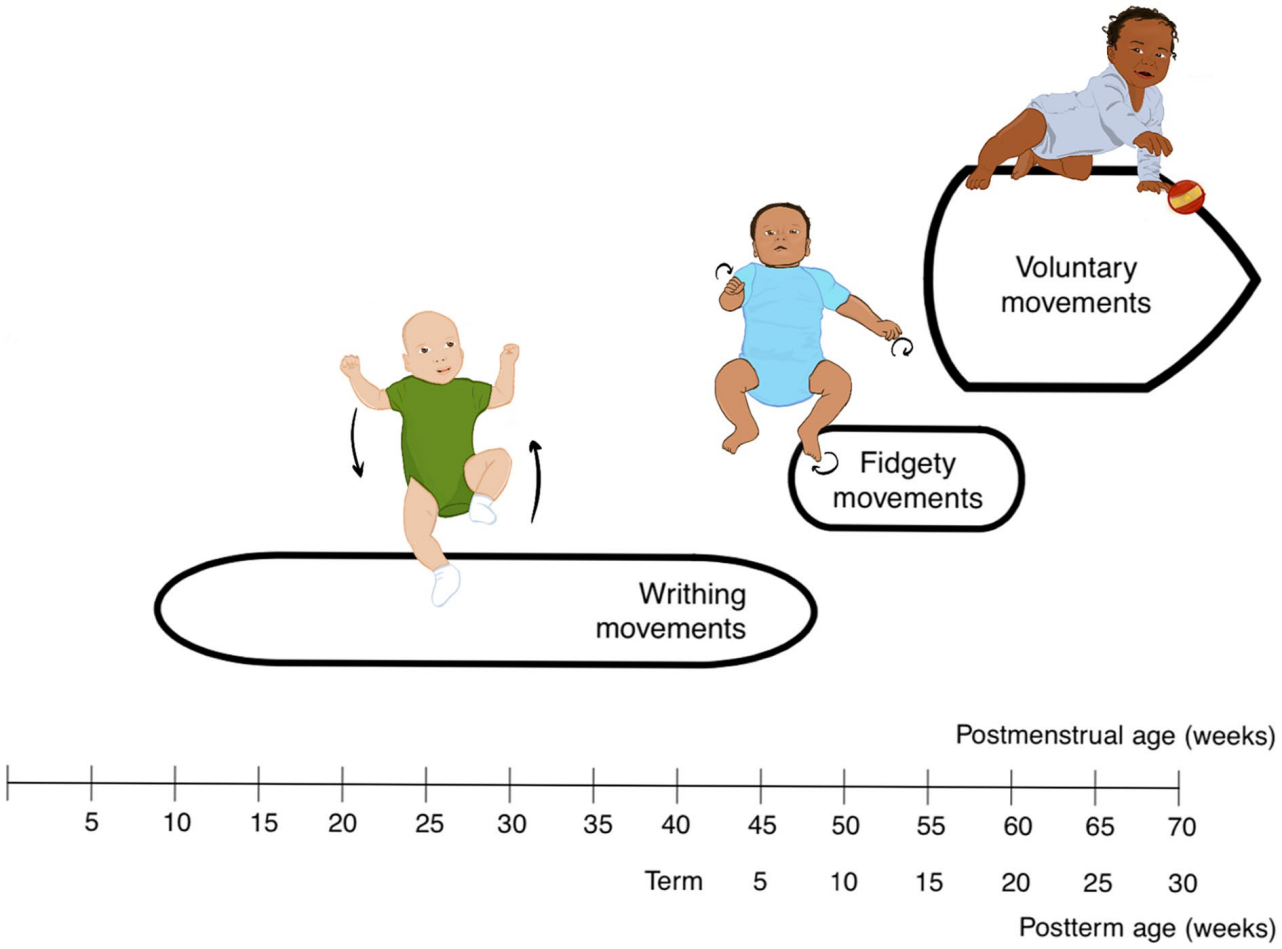
Timeline based on the developmental course of general movements (Prechtl, 2001).

Beyond the womb, the infant still produces GMs that can provide clinicians insight into the development of their nervous systems. GMs tend to follow a timeline of different movement patterns (Einspieler and Prechtl, 2004): writhing movements (lasting from prenatal stage till 8 weeks post-term age), fidgety movements (until 20 weeks post-term age) and finally, goal-directed/ voluntary movements. Writhing movements are elliptical full body movement sequences of the neck, limbs, and trunk. They are often described as a ‘swimming’ action with alternating arm and leg motion and have gradual onset with low-to-moderate speed and amplitude. Fidgety movements are more localized and appear as dance-like patterns or circular motions of the limbs with a small amplitude and moderate fluctuations in acceleration. Voluntary movements present with anti-gravity postures and orientation in space, where the infant may start to reach for objects and lift arms and legs upward, toward midline (Figure 2).

Heinz Prechtl’s general movements assessment (GMA; Prechtl, 1977, 1984; Einspieler and Prechtl, 2004) suggested that several aspects of the infant’s movements—absence of fidgety movements, chaotic GMs, poor repertoire, cramped-synchronized GMs are signs of neurodevelopmental derailment and decreased modulation of central pattern generators, CPGs. Variability is a crucial aspect to look for, in which the infant is able to move each limb and joint across various degrees of freedom (see Notes; Thelen and Smith, 1994). Furthermore, micro-analyses of film and sound in neonates (Condon and Ogston, 1966; Condon, 1970; Condon and Sander, 1974a,b) along with the use of wearables (Torres et al., 2016), have revealed very early disruption in neuromotor development, as have delayed neonatal reflexes (Teitelbaum et al., 1998, 2004).

In this literature review, we aim at providing a holistic understanding of the field of GMA, quantify its trends, forecast where it is headed and discern areas of improvement. Incorporating more quantitative and objective means of analysis, bibliometric methods were used to create networks of connections between various aspects of GMA-related publications, such as co-occurring key words, authors, and cited references.

We explore the dynamics of the methods of conducting the GMA, comparing the development of technological approaches to track general movement patterns quantitatively with clinical assessment by expert observation qualitatively. This notion of objectivity (bibliometrics) versus subjectivity (descriptive analyses) can also be seen in the field of systematic literature reviews. To better understand how these two methods can be compared and integrated to create a review, we first manually created a mind map of GMA literature in a manner like the ‘Zettelkasten method’, a common note-taking system to create an interconnected network of thoughts.

### Manual analysis

**Figure 3:**
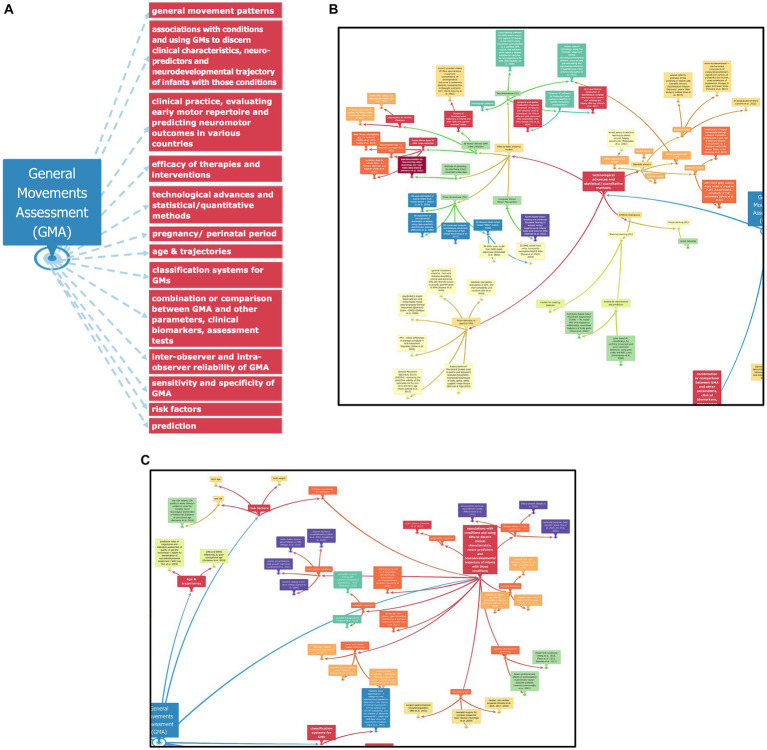
**(A)** Diagram outlining 13 distinct major themes/ key phrases observed from the literature collected in this study, organized by noting down summarizing information from each paper and then connecting them together to a broader, interconnected web. **(B)** Portion of the mind map, manually created with the ‘InfoRapid KnowledgeBase Builder’ tool, where different technological and statistical advances incorporated in the field of GMA can be seen. **(C)** Part of the mind map, which describes how GMA is used in various studies to predict medical conditions and measure developmental trajectories of infants and children, particularly those at high risk for neurodevelopmental delays.

While reading through the expanse of literature, 13 distinct themes were observed (see Figures 3A–C). Various aspects of GMA were observed such as functions of the assessment to predict conditions like cerebral palsy, to be used as a preliminary measure of neuromotor function for experimental studies, and to compare with other neurodevelopmental tests and clinical biomarkers or imaging techniques. In Figures 3B,C and Appendix Figure 1, referenced research papers for the topics and themes of the GMA map have also been indicated.

## Materials and methods

In creating the bibliometric networks, the dataset is sourced from academic research databases. A common limitation in systematic literature reviews is the size of the database. To resolve this, three prominent databases were used: SCOPUS, Web of Science (WoS) and Dimensions. These resources differ in their number and type of documents published. For instance, compared to SCOPUS and WoS, Dimensions provides a varied dataset that includes more recent pre-prints, conference papers and dissertations (Thelwall, 2018).

To find literature related to GMA, a search with the topic field: “general movements assessment” OR “general movement assessment,” as both forms are expressed, was performed on the databases. On Web of Science, search results were limited to the ‘Article’ and ‘Meeting Abstract’ document types. SCOPUS results were filtered to ‘Article’ and ‘Conference Paper’, and Dimensions to ‘Article’ and ‘Preprint’. Meeting abstracts, conference papers and preprints were incorporated to ensure the latest research could be considered and to provide insight into the future of the field of GMA. Reviews, editorials, books, commentary notes and letters were not included in order to avoid redundancy of mentioned research projects. The following search commands were input into the three database search engines:

SCOPUS: [TITLE-ABS-KEY (“general movements assessment”) OR TITLE-ABS-KEY (“general movement assessment”)] AND [LIMIT-TO (DOCTYPE, “ar”) OR LIMIT-TO (DOCTYPE, “cp”)].

WoS: “general movement assessment” (Topic) OR “general movements assessment”(Topic) AND Article OR Meeting Abstract (Document Types).

Dimensions: “general movements assessment” OR “general movement assessment” (Free text in title and abstract) AND Article OR Preprint (Publication Types).

**Figure 4:**
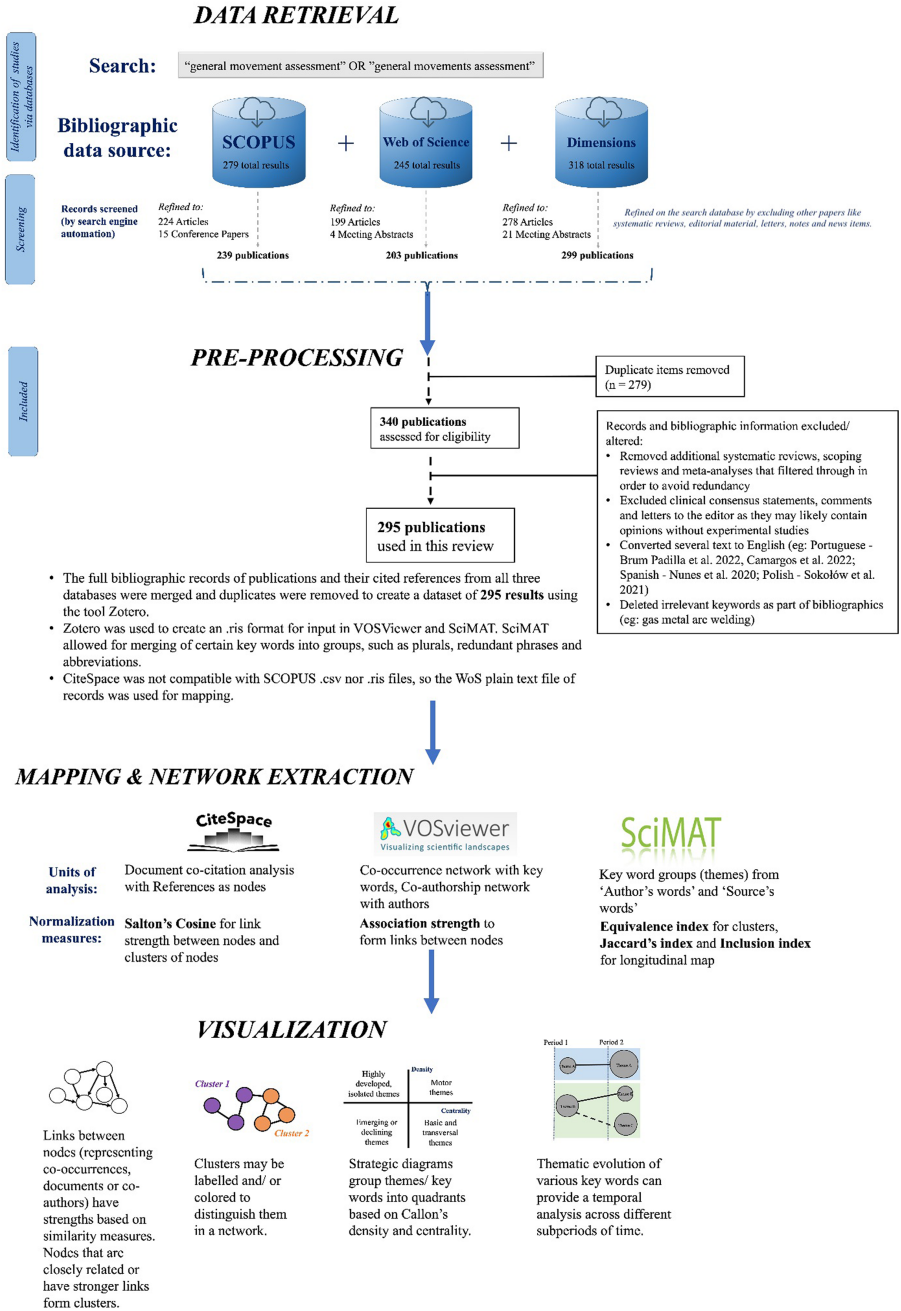
Flowchart of steps used to create bibliometric networks related to GMA, inspired by the Preferred Reporting Items for Systematic Reviews and Meta-Analyses (PRISMA) 2020 checklist.

From the corpus of research articles spanning from the year 2001 to 2023, bibliometric information was extracted—including publication title, author(s), abstract, journal, cited references, author and index keywords. SCOPUS, WoS Core Collection and Dimensions literature datasets were combined, and duplicates were merged, using Microsoft Excel and Zotero, a type of reference management software. Though the search results filtered most reviews, certain meta-analyses, systematic reviews, and editorial pieces were found and deleted in the following preprocessing steps (see Figure 4).

As seen in the steps listed in Figure 4, three bibliometrics software tools were used to create networks and strategic diagrams: VOSViewer (Van Eck and Waltman, 2007, 2010), CiteSpace (Chen, 2006) and SciMAT (Cobo et al., 2012). Each software tool differs based on statistical analyses and normalization measures used in the network maps as well as the types of networks/figures they can generate. For instance, while CiteSpace and SciMAT use set-theory based statistics to cluster and connect nodes, VOSViewer uses probabilistic measures of association strength between nodes.

Pre-processing of data involved removing duplicates and articles not related to GMA. Some article abstracts were also translated from various languages such as Portuguese and Spanish to ensure the keywords could be compared accurately in the bibliometric software. Synonymous words, pluralized/ singular forms of phrases, and abbreviated/ full form versions of author names were grouped, to prevent redundancy of terms. This process was carried out with a thesaurus file created for VOSViewer (see Appendix for additional information) and with the ‘word group manual set’ option on SciMAT.

### Automated software algorithm-based analysis

**Figure 5:**
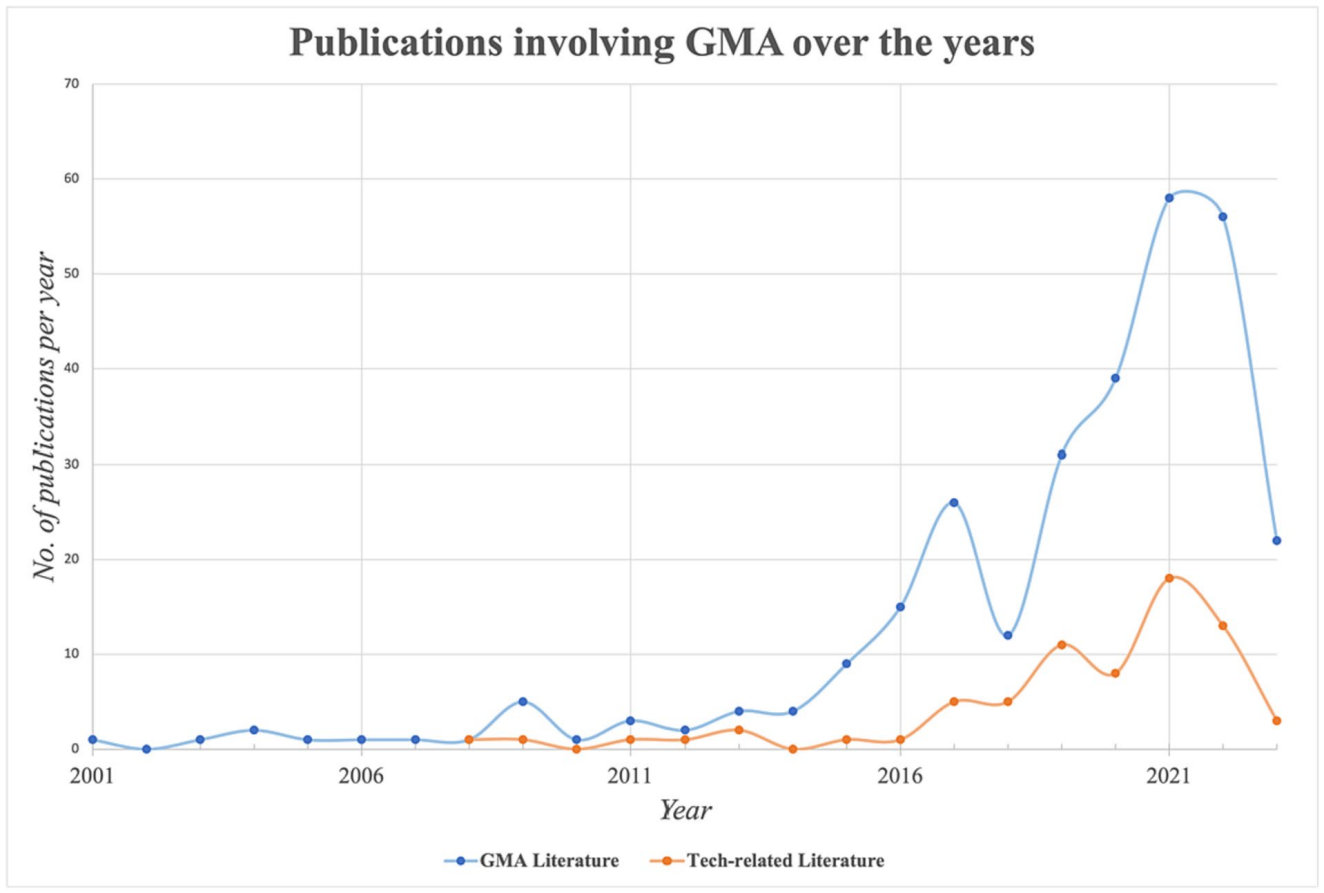
295 publications distributed over the years– sourced from WoS, Dimensions and SCOPUS; 71 articles were found to be tech-related, using digital biosensor means to quantify motion changes and their rates of change.

Over the years, 295 studies (excluding literature reviews and meta-analyses) have been published on the subject of GMA, as shown in Figure 5. In this paper, the focus on technological or sensor-based methods for GMA will also be investigated through bibliometric networks. From an overall dataset of 295 publications since 2001, 71 publications appeared to be related to this technology.

VOSViewer was used to create networks of co-occurring keywords present in publications and collaborations of co-authors. In VOSViewer, network visualizations of co-occurrences and co-authorship were created. Maps of co-occurring key words or phrases (co-occurrences) can show connections between prominent themes, methods and ideas extracted from titles, author keywords and abstracts of publications. For an overview of co-occurrence relationships, maps were created based on bibliographic data focused on keywords (author keywords, index keywords) and based on text data (publication title and abstract).

Each circular point or node of the co-occurrence network in two-dimensional space represents a key word present in the corpus of literature. The ties among nodes of the network have positive, numerical weight attributes. A weighted link between two co-occurring keyword nodes indicates that they have appeared together in publications, and in this case, the links have been assigned strengths by fractional counting measures, which ensures all the links connected to a node have weights fractionalized out of the total number of node-related links and add up to 1. Based on these weights, the nodes are assigned to various colored clusters as a measure of relatedness. Further, what distinguishes VOSViewer from other bibliometric software is its normalization of nodes and links with the probability-based measure of association strength. The association strength is proportional to the ratio of the numbers of observed co-occurrences to expected co-occurrences, assuming both occurrences are statistically independent (Eck and Waltman, 2009).

CiteSpace enabled the design of document co-citation analysis networks, which indicated relations between cited references. SciMAT created quadrants of Callon’s density and centrality, indicating thematic keywords with internal links and external links, respectively, to others in the literature. Elaborate and more detailed descriptions of creating bibliometric networks can be found in the Appendix, as a playlist series of tutorials.

## Results

### VOSViewer

**Figure 6:**
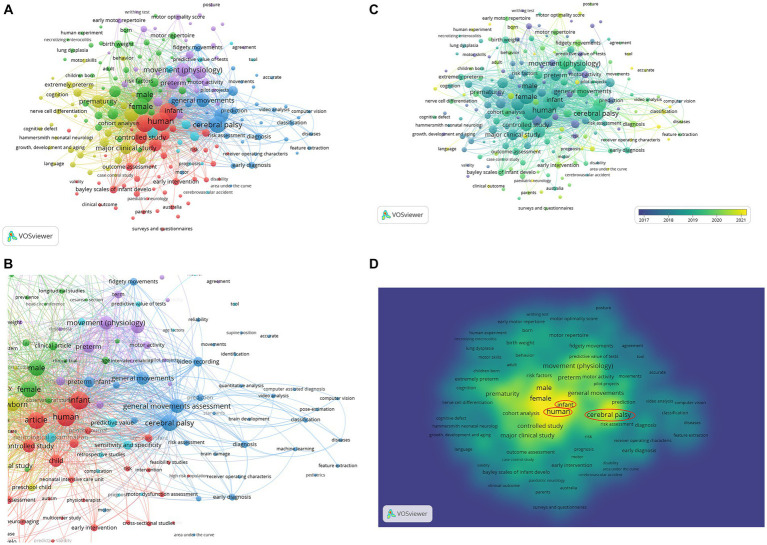
**(A)** VOSViewer network map of 197 co-occurrences, organized into six clusters. **(B)** Closer examination of the dark blue-coded cluster, with key words like ‘video recording’, ‘computer vision’, ‘deep learning’, ‘machine learning’, ‘quantitative analysis’ and ‘computer assisted diagnosis’ reveal the recent emergence of new digital methods. **(C)** VOSViewer map of 197 co-occurrences; overlay is color-coded by the average years of publication. **(D)** VOSViewer map of 197 co-occurrences, seen as a density visualization, with prominent keywords circled in red.

In the Figures 6A,B, a co-occurrence analysis was applied with the fractional counting method to form a connected network. The minimum number of co-occurrences of a keyword was set to 6— of the 2,189 total keywords found by VOSViewer, 197 met this threshold. The keyword ‘human’ had the highest number of occurrences, with a total link strength of 192.

Six different clusters were created, highlighting distinct themes of co-occurring keywords (Figure 6A). Cluster 1 is the largest and is shown in red, and it possesses nodes that have the largest number of occurrences (‘human’, ‘infant’, ‘article’). It illuminates clinical assessments and aspects of GMs, and in a broader sense, it shows various means of testing the function and presence of non-neurotypical signs of the developing nervous system. This cluster includes different assessments like ‘albert infant motor scale’, ‘bayley scales of infant development’ ‘hammersmith infant neurological examination’, ‘gross motor function classification’ and ‘motor dysfunction assessment’, which are often used in conjunction with GMA. These assessments are linked to words related to the experience of diagnosing infant patients, aiding with early intervention (see ‘physiotherapy’, ‘intervention’) and carrying out experiments (‘cross-sectional studies’, ‘randomized controlled trial’, ‘multicenter study’, ‘feasibility studies’, ‘article’, ‘priority journal’) in the clinical setting such as at neonatal intensive care units. A common theme has been the role of the parents and caregivers in these studies, focusing on the ‘child parent relation’, ‘high risk infant’, ‘care’ and ‘parents’, which are also associated with ‘follow-up’ studies that are able to incorporate GMA and other assessments to discern the developmental trajectory of the child. It is also interesting to note that observational assessments mentioned in this cluster utilize a pen-and-paper method in collecting data, in the form of ‘surveys and questionnaires’, ‘rating scale(s)’ and clinicians’ outcome assessments. This observational approach is utilized in the Autism Diagnostic Observation Schedule (ADOS) to assess autism spectrum disorder—among the pen-and-paper-based keywords and within the red cluster, the only neurological condition mentioned appears to be ‘autism’.

The green cluster, or cluster 2, also features themes from the clinical setting, but with a spotlight on factors (or more specifically, ‘risk factors’) that can alter the infants’ GMs and neurodevelopment. Aspects like ‘gestational age’, ‘birthweight’, ‘male’, ‘Apgar score’, ‘perinatal stage’, ‘female’, ‘head circumference’ and medical issues such as ‘lung dysplasia’, ‘brain hemorrhage’ and ‘necrotizing enterocolitis’ are all factors to consider when observing motor repertoire and early markers for dysfunction. Cluster 4, in yellow, is heavily intertwined in probabilistic space with clusters 1 and 2. It also features clinical assessments like the ‘hammersmith neonatal neurological examination’ and shows how ‘extremely low birthweight’ and ‘extremely preterm’ can relate to ‘motor performance’ and ‘cognition’. More prominently, the biological structure of the nervous system is a common thread among the yellow nodes, where ‘white matter’, ‘magnetic resonance imaging’ and ‘nerve cell differentiation’ can be used to improve our perception of developmental disabilities.

Cluster 5, seen in purple, centers movement (—‘movement’ in terms of physiology, ‘early motor repertoire’, ‘motor activity’ and ‘spontaneous motility’) and specific characteristics of the GMA. ‘Posture’ and ‘body position’ are important to discern while observing GMs in infants. The term ‘writhing test’ is seen closer to the ‘early motor repertoire’ node in the cluster, and writhing movements are generally used to learn more about the earliest stages of fetal/ infant motor activity and development. This cluster allows us to infer that ‘quality’ and ‘reproducibility’ from results of GMA are important amidst the presence of ‘observer variation’ and limitations of needing ‘agreement’ among assessment inter-raters or expert clinicians to produce a reliable neurodevelopmental outcome measure.

The smallest cluster, cluster 6 (cyan-colored), appears distributed widely across the network and within other clusters, and does not seem to have a distinct theme. This cluster has certain co-occurrences concerning metrics used to assess the outcomes and accuracy of screening and diagnostic tests, such as ‘sensitivity and specificity’ and ‘predictive value’.

Perhaps the most intriguing result is the dark blue cluster (cluster 3) protruding outward, relatively isolated from the rest of the clusters.

As seen in Figure 6B, cluster 3 topics related to digital methods of screening for infant neurodevelopment and GMA, such as ‘video analysis’, ‘pose-estimation’, ‘machine learning’, ‘feature extraction’, ‘computer vision’, ‘computer assisted diagnosis’, ‘quantitative analysis’ and ‘classification’ of certain GMs like fidgety movements. These nodes lie further away from themes directly centered on clinical assessments and indicate that these technological advances are not currently seen in the healthcare setting nor in medical studies and tests. Figure 6C highlights that the cluster has a more recent average age of publication compared to the others. Yellow nodes of ‘pose-estimation’, ‘classification’ and ‘machine learning’ show they occurred primarily in documents published in 2021. GMA has primarily been utilized to detect the presence of cerebral palsy (CP), as seen by the node within this cluster, and technology has been used to produce promising results in the detection of CP. Further, CP can be more easily detected as the GMs are more specifically defined and determined, for instance, by the absence of fidgety movements—this contrasts with detection for other conditions like autism spectrum disorder. The key words of ‘general movements’ and ‘general movements assessment’ also appear in this cluster, though positioned toward the center of the network.

Overall, cluster 3 appears highly specialized but does not have many occurrences nor strong total link strengths. Figure 6D illuminates this notion, as the kernel density of the computational methods is quite low compared to the central nodes like ‘human’, ‘cerebral palsy’ and ‘infant’. It is evident that there is a distinction in themes laterally as well. On the left side of the network, the nodes revolve around healthcare, or the clinical setting and measures carried out along with GMA. The right side (featuring the purple and dark blue clusters) showcases details of the field of GMA and those related to movement. Across all clusters, there is a re-occurring theme of prediction (see ‘predict’ in cluster 5, ‘prediction’ in cluster 3, ‘predictive validity’ in cluster 1 and ‘predictive value’ in cluster 6) which highlights the aim of most studies in creating tests that can accurately predict the neurodevelopmental outcomes for the infant, and hence, the need and types of supportive interventions.

**Figure 7:**
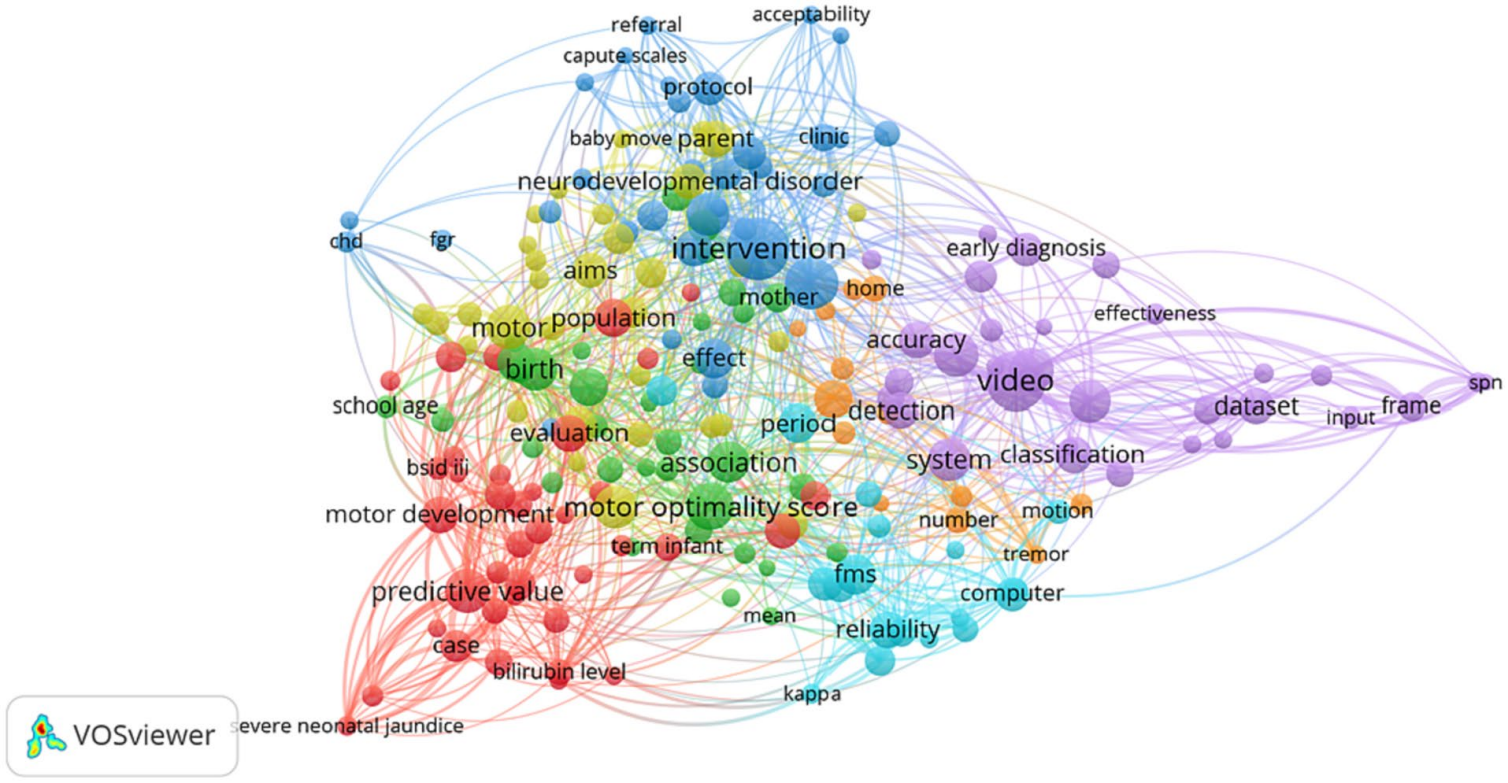
VOSViewer co-occurrence map using textual data from titles and abstracts of GMA-related publications.

For deeper insight into the methodology used in the expanse of publications, a VOSViewer network of term co-occurrences based on text data was created, seen in Figure 7. Regardless of keywords determined by authors and publishers, this map would parse and connect phrases from titles and abstracts, which contain a summary of the methods section. Of the 4,524 terms determined by VOSViewer, only 200 were selected with a minimum number of occurrences for each node being set to 7. Similar to Figure 6B, the purple cluster (cluster 5) in Figure 7 also extends outward, with distinct nodes focusing on technology associated with algorithmic models and pose-estimation like ‘classification’, ‘input’, ‘body parsing’, ‘body part’, ‘frame’, ‘feature’, ‘training’ of ‘model(s)’ and ‘data’.

**Figure 8:**
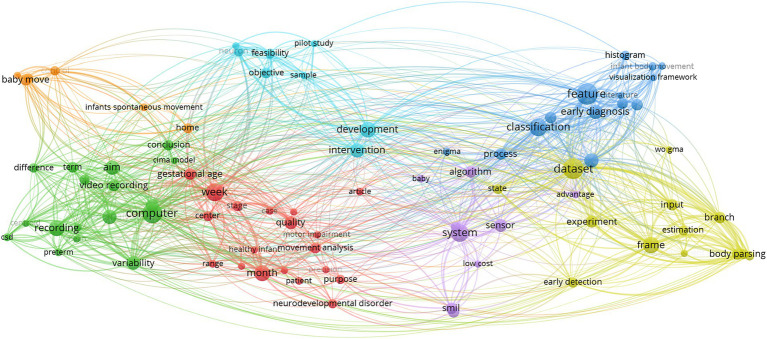
VOSViewer co-occurrence network with 86 items and 7 clusters, based on keywords from the titles and abstracts of tech-related publications.

Probing into what the technology cluster may entail and methods involved in these niche papers, a text-based map was also created from the corpus of 71 publications using digital approaches to detecting and testing GMs (see Figure 8). The clusters appear to be dispersed further away from one another compared to previous maps, where the terms related to the infant and data collection process (‘parent’, ‘baby move’ app, ‘home’, ‘infants spontaneous movement’, ‘video recording’, ‘recording’) lie on the left in the orange and green clusters while terms about digital data analysis (yellow cluster – ‘body parsing’, ‘frame’, ‘estimation’; blue cluster – ‘classification’, ‘feature’, ‘histogram’; and purple cluster – ‘algorithm’, ‘sensor’) are on the right.

**Figure 9:**
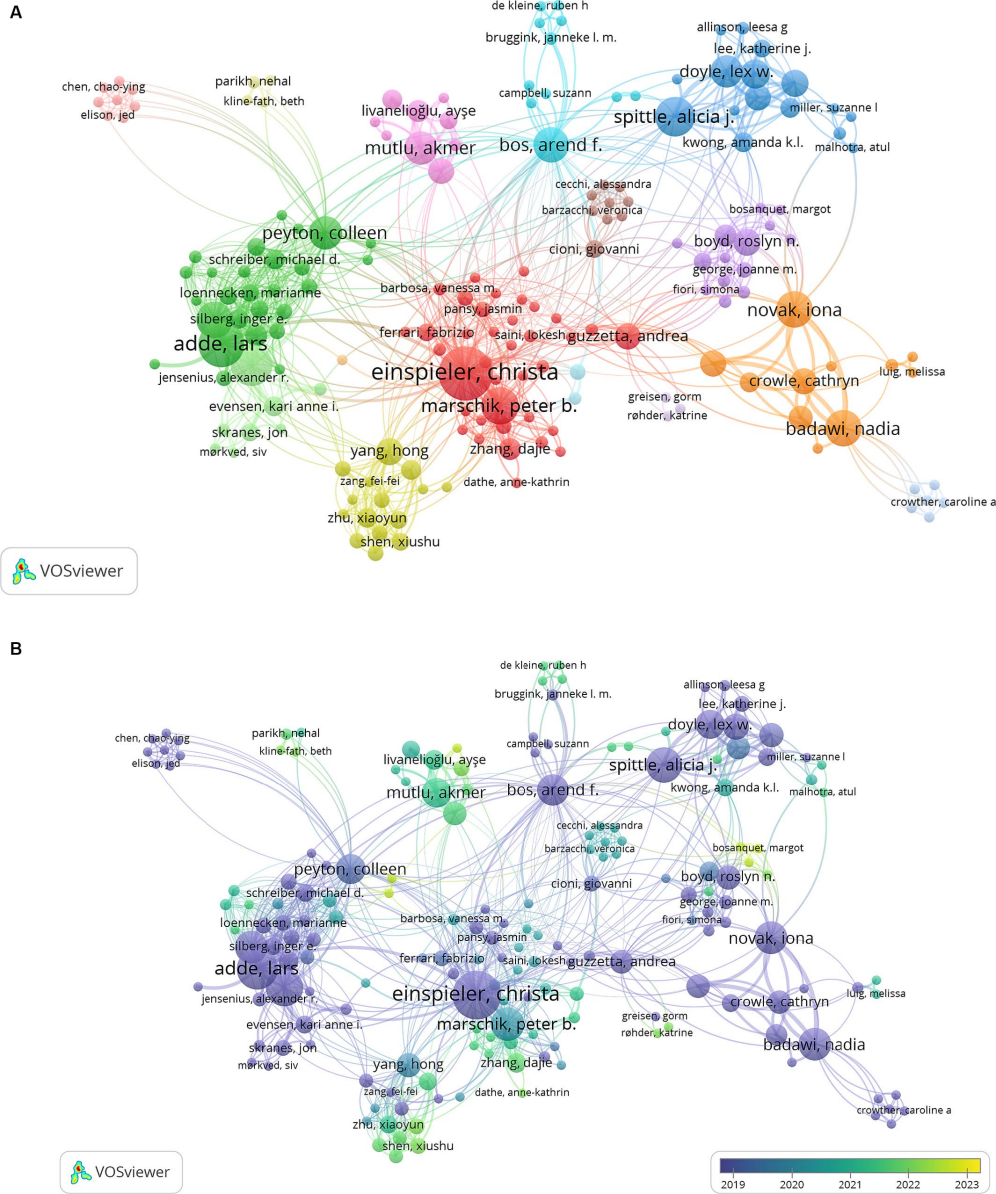
**(A)** VOSViewer network map of 181 publication co-authors, indicating 16 different clusters. **(B)** VOSViewer network map of 181 publication co-authors, in clusters and labeled with year of publications.

A co-authorship network from the publications was also created with VOSViewer, seen with Figures 9A,B. In Figure 9A, the network maps of co-authors display authors with a minimum of 2 document publications, having links weighted by fractional counting. Of the 262 authors in the network, the largest set of connected items was of 181—this connected set is seen in Figure 9A. It shows that the largest cluster (cluster 1, red) features neurophysiologist and pioneer of Prechtl’s GMA, Christa Einspieler (31 publications). In her cluster, developmental neuroscientists Peter Marschik and Andrea Guzzetta are also situated in the middle of the network, possessing strong external links. Clusters appear to closely connect researchers of similar field/background, universities, and location (red – Europe, pink – Turkey, orange – Australia). The largest author nodes in various clusters, like Christa Einspieler, appear to be of authors who are medical researchers and physicians that collaborate with interdisciplinary teams, like physical therapist Lars Adde, physician Nadia Badawi and occupational therapist Iona Novak, physiotherapist Alicia Spittle, pediatrician Arend Bos and physiotherapist Akmer Mutlu. There does not appear to be a lot of integration of engineers and computational scientists within the displayed connected set of authors. Authors publishing documents on specialized areas that mention GMA like magnetic resonance imaging (cluster 13 – including Nehal Parikh, Beth Kline-Fath), and transcranial magnetic stimulation to assess corticospinal connectivity and neuro-excitability (cluster 10 – Jed Elison and Chao-ying Chen) are seen as clusters extending away from the center. Figure 9B displays the co-authorship network with an overlay of the average years of publication. It appears that the authors with more central nodes, indicating their higher numbers of publications and collaborations, have average publication years of 2019–2020, while the authors at the periphery of the network have more recent publication years (2022–2023).

**Figure 10:**
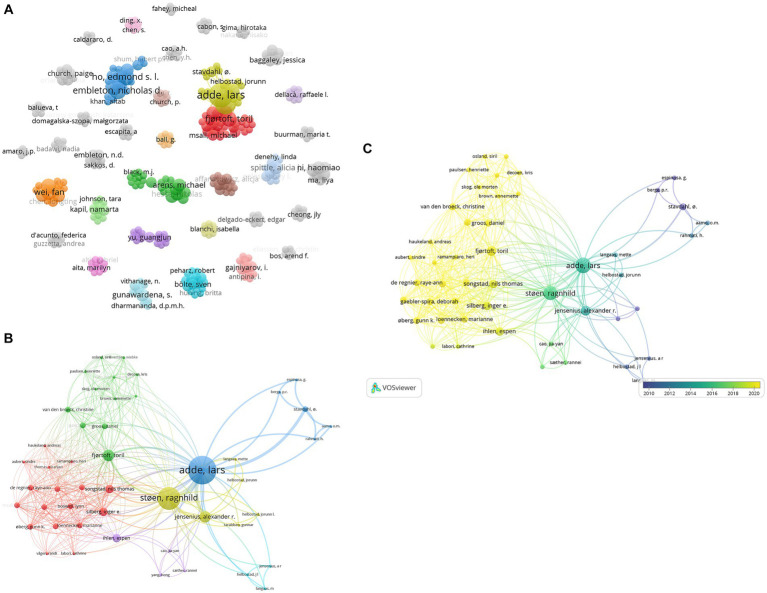
**(A)** Co-authorship network of literature related to technology-based methods of GMA, displaying 359 authors and 37 clusters. **(B)** Largest connected set within the co-authorship network seen in **(A)**, showing 48 authors grouped into 6 clusters. **(C)** Overlay visualization with average years of publication for authors in the largest connected set within the co-authorship network seen in **(A)**.

When focusing on the digital or technology-related publications, it is observed that the clusters of co-authors form sparsely populated isolated networks, seen in Figure 10A. Out of 359 authors (Figure 10A), only 48 of them form the largest connected and cohesive network. Toward the upper right portion of Figure 10A, the network containing the researcher node ‘Lars Adde’ is seen and it appears to be the largest interconnected set from this map. Figure 10B displays a version of the map with a closer view of this set of authors. With an overlay indicating average years of publication for Figures 10B,C shows recently published authors (average year ~2020) forming a more closely intertwined web toward the left of the map.

### CiteSpace

**Figure 11:**
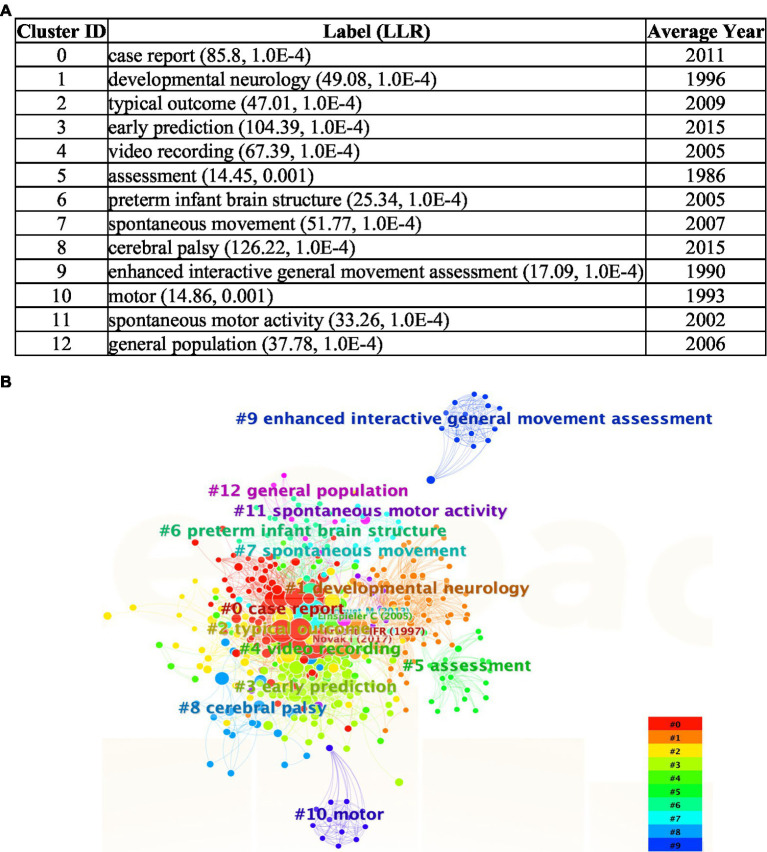
**(A)** Table of cluster themes, from CiteSpace document co-citation analysis network of 206 publications. **(B)** CiteSpace network map of clusters labeled #0–12, in ascending order of cluster size.

Using CiteSpace, a document co-citation analysis was carried out from 206 articles and meeting abstracts obtained from Web of Science, creating a network of clusters labeled automatically. These clusters are seen in the table in Figure 11A, where 12 major themes are present from the corpus of cited references related to GMA. Labels created based on log-likelihood ratios indicate that the cluster ‘case report’ has the greatest number of cited papers. As seen in Figure 11B, a network map was created, displaying the clustered co-citation web of key themes. In the ‘case report’ cluster, Novak et al., 2017 is the most cited paper (96 citations) followed by Prechtl et al. (1997) (91 citations) and Ferrari et al. (2004) (84 citations).

Clusters ‘#3 early prediction’ and ‘#8 cerebral palsy’ appear to have the most recent average year of all clusters— 2015, suggesting that the cited papers are progressing toward discerning earlier and more accurate diagnoses of neurodevelopmental conditions, particularly CP.

Similar to the VOSViewer map networks, CiteSpace co-citation clusters also show a distinct isolated branch related to technological aspects of GMA observation.

Cluster #9, ‘enhanced interactive general movement assessment’ or ENIGMA, is a computer-based software tool developed by Berge et al. to aid in General Movement expert knowledge elicitation and modeling.

### SciMAT

**Figure 12:**
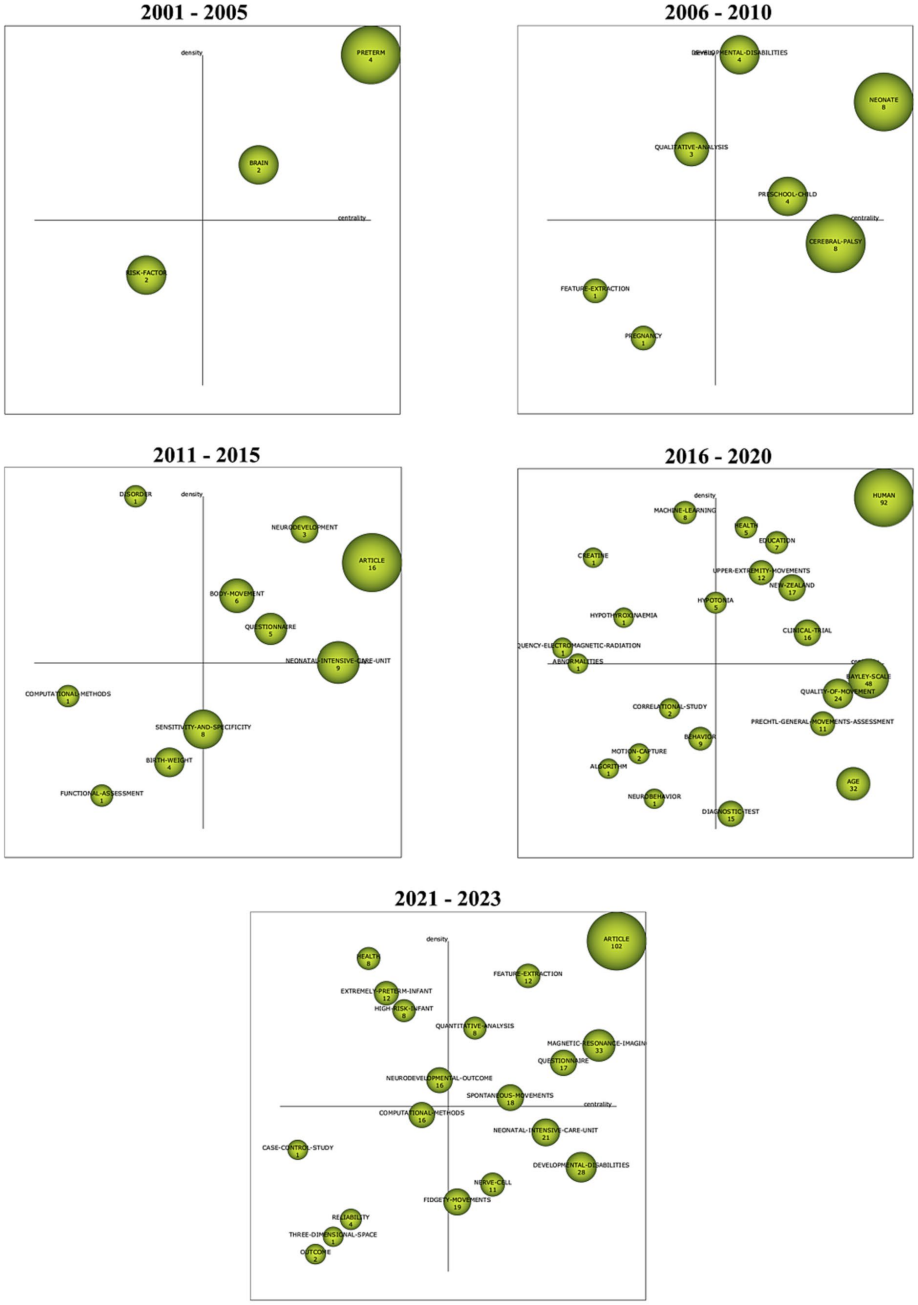
SciMAT strategic diagram of major themes in time periods ranging from 2001 to 2023 arranged according to Callon’s measures of density and centrality; the size of the nodes are based on the number of documents in which the keywords are present.

With SciMAT, the dynamic evolution of various themes or keywords can be mapped. Using quadrants as measures of Callon’s density and centrality, Figure 12 shows topics that have been specialized further with greater internal links (increases in density) and important across all publications with greater external connections (increases in centrality). Co-occurrence networks were created with the Equivalence index as the normalization measure. Jaccard index and Inclusion index were the evolution and overlapping measures, respectively.

The evolution of the major themes in the GMA begins with the keyword themes of ‘preterm’, ‘brain’ and ‘risk-factor’ (Figure 12). Researchers published papers in 2001–2005 focusing primarily on GMA in pre-term infants and what it can tell us about their nervous systems or brains. Other risk-factors like cerebral palsy, dyskinesia, psychomotor disorders, and neurological disorders (mentioned in the risk-factor cluster network, seen in the Appendix section) and their effect on the GMA appeared to be emerging themes in the literature. In this map, there are terms that relate to qualitative analyses and clinical assessment by human observation. During 2006 to 2010, the key word ‘qualitative analysis’ appears to have strong external links with other themes (high density) while terms related to computational methods like ‘feature extraction’, seen in the bottom left quadrant, are steadily emerging. Eventually, the themes of ‘computational methods’ and ‘machine-learning’ show relative increases in Callon’s density (2011–2015, 2016–2020). In 2021–2023, the themes of ‘feature-extraction’ and ‘quantitative analysis’ are motor themes and they have become central and influential in the field of GMA literature. The keyword ‘computational methods’ shows a significant increase in centrality along the *x*-axis, but remains on the left lower quadrant with low density. This denotes a steady appearance of computational methods in this corpus of literature (from 1 occurrence to 16 occurrences). The theme of ‘three-dimensional space’ lies further down the scales of Callon’s density and centrality. According to Callon’s analysis, this location forecasts that 3D video analyses or GMA of infants’ movement trajectories will likely continue as a trend.

## Discussion

The present work sets out to provide a literature review using new methods that enable rapid integration of a large corpus of papers on GMA. These new methods are now available in open access software that permits visualization of interconnected themes and subthemes across different research groups and their historical evolution. The outcomes of the analyses reveal patterns of relations among researchers and across themes of research. They further reveal trends within past research forecasting future tendencies as well.

Since the advent of bibliometrics methods, and to the best of our knowledge, this study is the first comprehensive analysis of work on GMA. In this sense, using these methods, we offer information about the historical evolution of the field and the emerging digital techniques that, according to the forecasting analysis, could potentially further advance it.

Manual analysis was also performed in Figures 3A–C where we see that along with being more time-consuming, it is evident that there are themes, particularly those related to technological advancements, that have been missed due to the vast expanse of literature in the field of GMA. There are however noticeable advantages to doing manual meta-analysis of the literature, despite it being time consuming. Expertise in a certain field enables research of themes that automated search may miss owing to restrictions in keywords across the literature on a particular subject. A manual search can be at times more detailed and informative of nuanced aspects of problems that keywords may not cover but that a human is fully aware of. Thus, the subjective element plays an important role that can now be complemented with the newly emerging computational bibliometric techniques.

Objective means of exploring papers and research-related information (such as author names, publication years, citations and journals) using statistical algorithms can prevent human-error and loss of relevant data when carrying out systematic reviews of the literature. The techniques featured here, and openly accessible to all, can illuminate historical patterns, forecast future trends and highlight groups of researchers playing key roles in the advancement of various aspects of the investigated themes. The automated computational connectivity analyses can, in this way, complement more traditional human-selected manual analyses that nevertheless remain very relevant to the overall understanding of basic scientific and clinical practices. To aid further utilization of these methods, we have created tutorials for researchers to use for their own literature searches and analyses (see 10.5281/zenodo.8103094), which can be part of a crucial first step in any research project or study.

Along the lines of clinical research, the results show a prevalence of signal detection theory in co-occurring keywords or themes of ‘receiver-operating characteristics’ and comparison of discrete scores made by observing clinicians (‘interrater reliability’). The prevalence of such methods is worrisome considering recent results from computational fields pointing at theoretical assumptions required by signal detection theory that are at odds with the empirical nature of infant’s developmental data (Torres et al., 2016). Indeed, new accounts of non-linearly accelerated growth and changes in movement variability longitudinally tracked in infants, point at a disconnect between *a priori* imposed theoretical assumptions of traditional analysis and the true empirical features of neurodevelopment. Computational trends uncovered in this literature analysis might offer new insights into the infant data digitally obtained by contemporary methods revealed in cluster 3 and emerging as recently as 2021.

VOSViewer maps shown in Figures 6A, 7 display such a recent cluster of nodes branching outward according to year of publication. This cluster represents digital means of assessing infant neurodevelopment, containing terms like ‘feature extraction’, ‘deep learning’, ‘computer vision’, ‘machine learning’ and ‘computer assisted diagnosis’. There does not appear to be more extensive connection and collaboration with these nodes to other themes. Further, this isolation of computer-aided analysis of GMA is seen through the co-authorship network where nodes of engineers and computational scientists are not well integrated with the other clusters of researching scientists and physicians. In addition, Figure 12 indicates that the theme of ‘computational methods’ in the lower left quadrant has increased slightly in density over the years (2011–2015 vs. 2021–2023), though it does not appear to have significant external links or centrality with other themes. Figure 11A also shows the CiteSpace co-citation network with an isolated theme of computational methods. In this sense, the results point at a need for interdisciplinary collaborations, e.g., between mathematicians, engineers or computational neuroscientists and clinicians, physiologists or developmental psychologists to address such a gap.

The bibliometric maps indicate that most GMA studies have been directed toward early prediction of neurological conditions and neurodevelopmental trajectories of the infant’s cognition and movement. Alongside clinical observation, the use of video analyses and biosensors to analyze movement patterns are emerging as a new, complementary way to achieve more reliable means of prediction and diagnoses, beyond what can be seen by the naked eye. These new technologies and algorithms can provide access to other types of infant spontaneous activity, such as orofacial movements during sucking patterns, as well as an efficient means of analyzing fetal activity through ultrasound imaging. The emergent methods will open a new window into neurodevelopmental disorders and help stratify them into clear-cut categories with far more precision than those indicated by clinical assessments alone—beyond a spectrum or continuum of conditions. Nuances captured by these technologies, beyond the limits of the naked eye, are bound to revolutionize the ways in which we screen, diagnose and track disorders of the nervous system.

The new, emerging computational methods aimed at complementing clinical observation of GMs will open the clinical space to continuous, dynamic analysis of stochastic video data in infant neurodevelopment. In turn, this level of precision and higher sampling frequency with commercially available means (e.g., smartphones and tablets) will push the field toward more scalable solutions to uncover new patterns across previously under sampled sectors of the population. Then, empirical validation of automated, briefer methods will begin the path of bypassing current assumptions of linearity, stationarity and normality in neurodevelopmental biorhythmic data that has been otherwise, empirically characterized as non-linear, non-stationary, non-normally distributed in modest sample sizes of neonatal cohorts (Torres et al., 2016; Chambers et al., 2020). Scaling such approaches will open new lines of inquiry in the field.

In summary, we have demonstrated the utility of several bibliometric methods in identifying and visualizing patterns that reveal historical patterns, forecast future trends, and uncover new emerging approaches, highlighting the need to collaborate and integrate multiple sources of clinical and computational data. When the field integrates human observation and digital means, the outcome will be much more than the sum of its individual parts. This change may be around the corner, according to the predictions of these analytical bibliometric techniques.

## Data availability statement

The original contributions presented in the study are included in the article/Supplementary materials, further inquiries can be directed to the corresponding author.

## Author contributions

HV and ET contributed to the conception and design of the study. HV organized the database, performed the statistical analysis, created all figures and tutorials, and wrote the first draft of the manuscript. ET edited the manuscript, and supervised and funded the study. HP, PK, and AG contributed to clinical insights. All authors contributed to the article and approved the submitted version.
